# Review of the use of nasal and oral antiseptics during a global pandemic

**DOI:** 10.2217/fmb-2020-0286

**Published:** 2021-01-19

**Authors:** Christopher Stathis, Nikolas Victoria, Kristin Loomis, Shaun A Nguyen, Maren Eggers, Edward Septimus, Nasia Safdar

**Affiliations:** 1 HHV-6 Foundation, Santa Barbara, CA 93108, USA; 2 Department of Otolaryngology, Head & Neck Surgery, Medical University of South Carolina, Charleston, SC 29425, USA; 3 Prof Dr G Enders MVZ Laboratory & Institute of Virology, Infectious Diseases, Stuttgart, BW 70193, Germany; 4 Department of Population Medicine, Harvard Medical School & the Harvard Pilgrim Healthcare Institute, Boston, MA 02215, USA; 5 Division of Infectious Disease, Department of Medicine, University of Wisconsin School of Medicine & Public Health, Madison, WI, USA & The William S Middleton Memorial Veterans Hospital, Madison, WI 53726, USA

**Keywords:** chlorhexidine, COVID-19, hydrogen peroxide, hypertonic saline, iota-carrageenan, nasal spray, oral rinse, povidone-iodine, respiratory infections, SARS-CoV-2

## Abstract

A review of nasal sprays and gargles with antiviral properties suggests that a number of commonly used antiseptics including povidone-iodine, Listerine^®^, iota-carrageenan and chlorhexidine should be studied in clinical trials to mitigate both the progression and transmission of SARS-CoV-2. Several of these antiseptics have demonstrated the ability to cut the viral load of SARS-CoV-2 by 3–4 log_10_ in 15–30 s *in vitro*. In addition, hypertonic saline targets viral replication by increasing hypochlorous acid inside the cell. A number of clinical trials are in process to study these interventions both for prevention of transmission, prophylaxis after exposure, and to diminish progression by reduction of viral load in the early stages of infection.

## Tweetable abstract

Commonly used antiseptics including povidone-iodine, Listerine^®^, iota-carrageenan and chlorhexidine should be studied in clinical trials to mitigate the progression and transmission of SARS-CoV-2.

The nose is both a source of pathogens and a critical port of entry for infectious agents such as viruses and bacteria. Proper hand hygiene is an essential step in infection prevention and control, but the nose and throat, critical portals of entry for infectious germs, are often not addressed. In terms of the current pandemic, some evidence indicates that viral titers of SARS-CoV-2 are extremely high in the nose and throat [1]. The hACE2 receptor, used by SARS-CoV-2 to establish infection, is found to be highly expressed in the mucosa of the oral cavity and nasal tissues [2].

Many preparations of antiseptics such as povidone-iodine (PVP-I), alcohol with essential oils, chlorhexidine (CHX), hydrogen peroxide (HP) and iota-carrageenan (IC) are widely used and safe for application to the epithelium. Hypertonic saline has also been investigated as an intervention against respiratory infections. WHO Interim guidance for dental practices recommends asking patients to rinse their mouths with 1% hydrogen peroxide or 0.2% PVP-I for 20 s prior to examination or starting any procedure for the purpose of reducing the salivary load of oral microbes, including SARS-CoV-2 [3].

This review explores common and/or promising antiseptic techniques and some of the ongoing clinical trials that are investigating the use of these antiseptic compounds as potential treatments and preventative measures.

## Materials & methods

A preliminary search for this review was conducted in PubMed using the search terms ‘nasal antisepsis’ and ‘throat antisepsis’ paired independently with ‘antiviral’ and ‘virus’. Based on the result of that initial search, an additional search was performed pairing the terms ‘povidone-iodine gargle’, ‘betadine gargle’, ‘hypertonic saline gargle’, ‘Nozin’, ‘chlorhexidine gargle’, ‘hydrogen-peroxide gargle’, ‘dequonal’, ‘dequalinium chloride’ and ‘benzalkonium chloride’ independently with ‘antiviral’ and ‘virus’. Articles from these searches, as well as articles referenced within the resulting articles, were used to identify key search terms for a more systematic search.

Based on the preliminary search, a new search was conducted in PubMed using the following search terms: (SARS-CoV-2 OR Covid-19 OR coronavirus OR SARS-CoV OR middle east respiratory syndrome coronavirus (MERS-CoV) OR SARS OR MERS) AND (nasal OR throat OR oral) AND (antimicrobial OR mouthwash OR rinse OR gargle OR antisepsis OR antiviral OR virucide OR biocide OR antiseptic prophylaxis) AND (catechin OR cyclodextrin OR citrox OR quaternary ammonium compounds OR cetrimide OR octenidine dihydrochloride OR dequonal OR benzalkonium chloride OR dequalinium chloride OR sage extract OR sodium hypochlorite OR PVP OR Nozin OR chlorhexidine OR cetylpyridinium chloride OR ethanol OR hydrogen peroxide OR hypertonic saline OR carrageenan). This search resulted in 47 individual results. The resulting studies were then fully read by two co-authors and additional papers were added after reference mining. Compounds were excluded if they were not commercially available. The only class of compounds that were commercially available but were excluded were the quaternary ammonium compounds due to safety concerns identified during the preliminary search.

A final search was performed of the clinicaltrials.gov database using the search terms (nasal spray OR gargle) AND SARS-CoV-2 to identify relevant clinical trials. A total of 20 unique results were identified, and trials were excluded based on the following criteria: non-randomized controlled trials, trials with fewer than 20 participants.

## Results

### PVP-I (betadine)

PVP-I is one of the most common antiseptics available and is considered safe to use on the mucosal epithelium at appropriate concentrations [4]. For the skin, PVP-I typically comes in a 10% preparation as well as a 1% preparation for the oral cavity, and is available in many countries including Japan, Australia and Canada [5]. Eggers *et al.* reported at a 0.23% dilution of 7% PVP-I mouthwash reduced viral titers of two coronaviruses similar to SARS-CoV-2, SARS-CoV and MERS-CoV, by over 4log_10_ after a 15 s exposure *in vitro* [6]. A list of the compounds and their effects on SARS-CoV-2 *in vitro* can be found in Table 1, and their effects on viruses other than SARS-CoV-2 can be found in Supplementary Table 1. In a clinical setting, gargling with PVP-I solution resulted in a 50% drop in respiratory infections caused by pathogens such as *Pseudomonas aeruginosa, Staphylococcus aureus* and *Haemophilus influenzae* [7]. Against SARS-CoV-2, *in vitro* testing of PVP-I at concentrations ranging from 10.0 to 0.45% were reported to yield a greater than 4.00 log_10_ reduction in viral titers after only a 30 s exposure [8]. PVP-I was also shown to have rapid inactivation of SARS-CoV-2 *in vitro* at concentrations of 1.5–0.5% after a 15 s exposure time with a 3.0 log_10_ reduction in viral titers [9]. In a preprint, *in vivo* toxicity study of PVP-I revealed no toxicity concerns with sub-chronic intranasal use of PVP-I [10]. PVP-I may be tolerable as not only an oral mouthwash but also as a nasal spray [11]. In one *in vivo* study of the effects of PVP-I on SARS-CoV-2 viral load, there was a decrease in viral load for two out of four patients after a 15 ml oral rinse of 1.0% PVP-I for 1 min [12]. A clinical study in 2007 showed PVP-I nasal spray at concentrations of 4.4 and 2.2% have shown no damage to the epithelium of the nasopharynx [5]. 3M has developed a PVP-I nasal swab for decolonization that includes a film forming polymer that increases persistence to 12 h [13]. Nasal PVP-I solution was more tolerable than traditional mupirocin nasal wash as a method of reducing *S. aureus* prior to surgery [14]. No true allergy to PVP-I has been noted, but a small percentage may develop low level skin irritation [15].

**Table 1.** *In vitro* effects of commercial nasal and oral rinses against SARS-CoV-2.

Compound	Concentration/product	Exposure	Viral titer reduction (log_10_)	Ref.
PVP-I	Throat Spray 0.45%	30 s	≥4.00	[8]
	Gargle/mouthwash 1.0%	30 s	≥4.00	
	Gargle/mouthwash 1.0%, 1:2 dilution	30 s	≥4.00	
	Oral rinse antiseptic 1.5%	15 s	3	[9]
	Oral rinse antiseptic 0.75%	15 s	3	
	Oral rinse antiseptic 0.5%	15 s	3	
	0.90%	30 s	3.5	[10]
	0.50%	30 s	3.2	
	0.28%	30 s	2.2	
	0.09%	30 s	1.2	
	1.25%	30 s	3.33	[16]
		15 s	3.0	
Ethanol	70%	30 s	3.33	[16]
		15 s	2.17	
	20%	30 s	1.1	[17]
	30%	30 s	≥5.9	
Ethanol and essential oils (Listerine Cool Mint)	(Ethanol 21%, menthol 0.042%, thymol 0.064%, methyl salicylate 0.06%, eucalyptol 0.092%)	30 s	>3.11	[18]
		30 s	>2.78	
		30 s	>2.61	
Chlorhexidine	Chlorhexidine 0.2%	30 s	1	[18]
		30 s	0.78	
		30 s	1.17	
	Chlorhexidine 0.2%	30 s	0.5	[18]
		30 s	0.56	
		30 s	0.5	
Hydrogen peroxide	1.5% stabilized hydrogen peroxide, glycerin and erythritol	30 s	0.78	[18]
		30 s	0.61	
		30 s	0.33	
Iota-carrageenan and xylitol	600 μg/ml (IC) + 50 mg/ml (X)	2 h pretreatment of Vero E6 cells	>4.25	[19]
	60 μg/ml (IC) + 50 mg/ml (X)		>4.25	
	6 μg/ml (IC) + 50 mg/ml (X)		>4.25	

IC: Iota-carrageenan; PVP-I: Povidone-Iodine; X: Xylitol.

Several clinical trials are underway that compare PVP-I at different concentrations and other antiseptics. In one of these studies, PVP-I 0.20% will be used as a gargle and nasal lavage for 20–30 s three times a day for 6 days [20]. This trial will also compare PVP-I to hydrogen peroxide, neem extract (an herbal antimicrobial) and hypertonic saline to elucidate which (if any) treatment reduces intraoral viral load. This safety of PVP-I as a gargle or nasal spray is assumed and will not be evaluated.

There is some evidence that betadine, a commercially available form of PVP-I, may decrease mucociliary clearance at a concentration of 5–10% by inhibiting ciliary beating of nasal epithelial cells in the sinonasal mucosa, indicating possible cilial toxicity [21]. However, there have been other papers with betadine products that demonstrate that there is no ciliotoxicity of PVP-I products [5,22].

There is also concern that PVP-I may have unknown effects on the microbial flora of the oral cavity [23]. PVP-I showed no deleterious effect on a representative nitrate-reducing species called *Veillonella dispar* in the oral cavity suggesting it may not have an effect on the normal oral microbial flora [24].

### Iota-carrageenan

Iota-carrageenan is a sulphated polysaccharide purified from red marine algae and has been shown to inhibit rhinovirus, influenza A and multiple herpesviruses *in vitro* [25,26]. In one preprint study, iota-CARRAGEENAN with xylitol inhibited SARS-CoV-2 by a reduction factor of >4.25 log_10_ *in vitro* at iota-carrageenan concentrations of 600, 60 and 6 μg/ml, with a xylitol concentration of 50 mg/ml [19]. In a randomized controlled trial, iota-carrageenan nasal spray was tolerable in patients with the common cold and had a good safety profile [27]. Xylitol itself has also demonstrated antiviral properties [28].

### Hypertonic saline

Several studies support the use of hypertonic saline as a preventative measure against respiratory viruses and there are already commercially available hypertonic saline rinses in many countries. Nasal saline rinses are well tolerated and there is evidence that they decrease the severity of upper respiratory tract infections [29].

Several randomized controlled trials have shown the efficacy of salt water in acute upper respiratory tract infections [30]. A small randomized controlled trial in the UK reported that saline irrigation and gargling reduced the duration of viral upper respiratory tract infections by 1.9 days (p = 0.01), reduced the transmission to household contacts by 35% (p = 0.006), reduced the use of over the counter medications by 36% (p = 0.004). More individuals in the intervention arm reduced viral shedding by >0.5 log_10_/day compared with controls (p = 0.04) [31]. The mechanism for the efficacy of saline against various viruses in the upper respiratory tract is that increased availability of local chloride ions (from NaCl) supports the production of hypochlorous acid (the active ingredient in bleach), a potential innate immune mechanism in epithelial cells [30,32]. The addition of NaCl to the cell culture medium inhibited various human viruses in a dose-dependent manner. Human coronavirus 229E replication was inhibited by saline concentrations between 30 and 50 mM [32]. Importantly, the entry of Cl^-^ ion into the cells and its conversion by a peroxidase to hypochlorous acid was found to be necessary for the observed antiviral activity [32]. The data did not support any other mechanism (i.e., virucidal, adsorption inhibition). A study investigating the effect of hypertonic saline on SARS-CoV-2 *in vitro*, available on the preprint server, showed that 210 mM NaCl (1.2%) inhibited virus replication by 90% (p < 0.0005) and 260 mM NaCl (1.5%) inhibited viral replication by 100% (p < 0.0005) in Vero CCL-81 cells when compared with 110 mM NaCl (0.6%) [33]. The concentration of NaCl in the mucus layer found in the respiratory tract measured by *in vivo* tests has been reported to be in a range of 110 mM (0.6%)–130 mM (0.76%) [34,35]. Any NaCl solution with a concentration >130 mM that is applied to the nasopharynx would result in a net increase in chloride ions and sodium ions in the mucus layer.

### Alcohol & essential oils

In one German *in vitro* study, 30 s of exposure to 78% alcohol reduced SARS viral loads by ≥5 log_10_, effectively inactivating the virus [36]. In another *in vitro* study investigating WHO-recommended formulations of virucidal agents, 30 s of exposure to 20% ethanol reduced SARS-CoV-2 infectivity by 1.1 log_10_ and a 30 s exposure to 30% ethanol reduced infectivity by ≥5.9 log_10_ [17]. The active compounds of Listerine^®^, a widely used oral rinse, that includes ethanol as an inactive ingredient with essential oils; eucalyptol, menthol, methyl salicylate and thymol has been found to significantly reduce virus activity [18]. In addition to ethanol, the essential oils each have independent antimicrobial and anti-inflammatory properties [37]. In quantitative suspension studies, conducted by researchers in Germany, several SARS-CoV-2 strains showed log reduction factors of 2.61–3.11 after being incubated in the Listerine Cool Mint formulation (21% ethanol) for 30 s [18].

Nozin Nasal Sanitizer is an ethanol-based nasal antiseptic commonly used in hospitals and surgical units that is reported to reduce median *S. aureus* units by 99% [38]. The product consists of 60–70% ethanol with jojoba and vitamin E to moisturize the nose, and it is applied around the inside of the vestibular surface of the nostrils using a sterile swab.

A Listerine product consisting of eucalyptol 0.092%, menthol 0.042%, methyl salicylate 0.060% and thymol 0.064%, in 25% ethanol has been reported to have no detrimental effect to the representative nitrate-reducing bacteria *V. dispar* of the mouth [24]. No toxicity, or deleterious effects on the oral microbiota have been reported for occasional use.

### Chlorhexidine

CHX mouthwash, widely used for gum disease, demonstrates bactericidal and virucidal activity against enveloped viruses such as coronaviruses [6,39,40]. The bactericidal activity is caused by the nonspecific binding of the positively charged chlorhexidine molecule to the membrane phospholipids of bacteria [41]. Some evidence indicates CHX is less effective in rapid elimination of human coronaviruses compared with other virucidal agents such as alcohols and PVP-I [42], but it also binds to proteins on the skin which expands the duration of protection up to 24 h [43]. One study in South Korea reported in two patients, there was a decrease in SARS-CoV-2 viral load for 2 h following the use of 15 ml of 0.12% chlorhexidine gluconate (CHG) mouthwash based on real-time PCR [44]. Chlorhexidine has been reported to be safe in the nasopharynx as a gel but there has not been much study of chlorhexidine as a nasal spray [45].

### Hydrogen peroxide

A 0.5% solution of accelerated hydrogen peroxide was able to reduce infectivity of human coronavirus 229E by greater than 4log_10_ after 60 s exposure in suspension tests [46]. Accelerated hydrogen peroxide also contains other ingredients such as surfactants that may contribute to its effectiveness so it is not clear that hydrogen peroxide alone was effective. A randomized trial of 68 intensive care unit patients reported that those whose oral cavities were washed with 3% hydrogen peroxide were significantly less likely to develop ventilator-associated pneumonia compared with controls who gargled saline [47]. Washes were performed by hospital staff using cotton swabs soaked in 15 cc of 3% hydrogen peroxide. Hydrogen peroxide mouthwash in one study reduced viral titers in three separate strains of SARS-CoV-2 but the reduction was not significant [18]. In one clinical pilot study of ten SARS-CoV-2 positive patients hydrogen peroxide 1% was not shown to reduce intraoral viral load [48].

### Clinical trials

Currently, there are over 20 trials testing the efficacy and tolerability of PVP-I, Listerine, iota-carrageenen, hypertonic saline, chlorhexidine, baby shampoo and hydrogen peroxide (see Table 2). We found 17 clinical trials that met our criteria. There is currently a systematic review being done by the Cochrane review of clinical trials for SARS-CoV-2 [23].

**Table 2.** Selected ongoing clinical trials of antiviral oral and nasal rinses against SARS-CoV-2.

Clinical trial #	Compounds tested	Use	EST (n)	Country	Start date	Ref.
NCT04371965	PVP-I	Gargle or nasal spray	24	France	Q3 20	[49]
NCT04341688	PVP-I, HP, neem extract and HS	Nasal lavage and gargle	50	Pakistan	Q3 20	[20]
NCT04347954	PVP-I or isotonic saline	Nasal spray	45	USA	Q3 20	[50]
NCT04410159	PVP-I, Listerine or tap water	Gargle	20	Malaysia	Q3 20	[51]
NCT04521322	IC	Nasal spray	400	Argentina	Q3 20	[52]
NCT04364802	PVP-I	Nasal spray and gargle	250	USA	Q2 20	[53]
NCT04352959	Beta-cyclodextrin and citrox	Gargle	206	France	Q2 20	[54]
NCT04449965	PVP-I	Nasal rinse and gargle	81	Canada	Q3 20	[55]
NCT04344236	Saline, PVP-I or CHX	Nasal rinse and gargle	48	USA	Q2 20	[56]
NCT04409873	Distilled water, HP, CPC, chlorine dioxide or Listerine (zero alcohol)	Gargle	150	USA	Q4 20	[57]
NCT04478019	PVP-I + CHG	PVP-I nasal swab + CHX gargle	94	USA	Q3 20	[58]
NCT04549376	PVP-I	Nasal irrigation	200	Bangladesh	Q3 20	[59]
NCT04393792	PVP-I or normal saline	Nasal rinse and gargle	40	UK	Q2 20	[60]
NCT04563689	CHG or CPC	Gargle	24	Colombia	Q2 20	[61]
NCT04337918	NORS	Nasal spray and gargle	200	Canada	Q2 20	[62]
NCT04537962	CHX, HP, CPC, zinc lactate	Mouthwash	70	Brazil	Q2 20	[63]
NCT04443868	NORS or saline	Nasal spray and nasal irrigation	300	Canada	Q3 20	[64]

CHG: Chlorhexidine gluconate; CHX: Chlorhexidine; CPC: Cetyl peridinium chloride; HP: Hydrogen peroxide; HS: Hypertonic saline; IC: Iota-carrageenan; NORS: Nitric oxide releasing solution; PVP-I: Povidone-iodine.

One clinical trial that has been completed at the Universiti Sains Islam Malaysia and has results for 20 participants is investigating the use of a PVP-I gargle or Listerine gargle on early viral clearance of SARS-CoV-2 defined as two negative real-time PCR results at least 24 h apart [51]. One clinical trial is investigating the efficacy of iota-carrageenan nasal spray use four times per day for 21 days as a preventative measure in healthcare workers [52]. The SHIELD study in the USA at the University of Wisconsin, Madison, is investigating the preventative efficacy against SARS-CoV-2 of a combination of PVP-I nasal swabs and chlorhexidine oral rinse [58]. A pilot study sponsored by Colgate Palmolive and Hospital Israelita Albert Einstein are investigating different formulations of Colgate^®^ Peroxyl^®^ hydrogen peroxide mouthwash and Colgate Periogard^®^ chlorhexidine oral rinse to determine effect on SARS-CoV-2 viral load [63].

NYU Langone is sponsoring a clinical trial that compares saline oral/nasal rinse to PVP-I and CHX in patients with a positive COVID-19 test [56]. For each potential treatment, participants will do a 5 cc nasal rinse in each nostril followed by a 20 cc oral gargle four times per day for 7 days. The viral load of SARS-CoV-2 will be measured by using cycle time to PCR as a proxy for viral load.

Vanderbilt University Medical Center is conducting a clinical trial of two interventions, saline nasal irrigation twice daily and normal saline with 1/2 teaspoon of baby shampoo nasal irrigation twice daily [65]. The primary outcomes measured will be change in SARS-CoV-2 mucosal immune response measured using viral RNA, change in microbial load in the nasopharynx and viral load in the nasopharynx based on quantitative PCR [65]. Based on the interim analysis of this study, nasal congestion and headache symptoms resolved 7–9 days earlier in the intervention groups compared with the no intervention group [66].

The University of Edinburgh is also conducting a clinical trial investigating the efficacy of hypertonic saline in patients with clinically suspected or confirmed COVID-19 [67]. The primary outcome of the study is time to resolution of symptoms.

Stanford HealthCare is conducting a clinical trial investigating PVP-I nasal sprays compared with normal saline nasal sprays [50]. In the trial patients with a COVID-19 diagnosis and a positive lab test will receive either 2, 0.5% PVP-I or isotonic saline 0.9% nasal spray as two sprays in each nare from a nasal spray bottle, four times per day. Change in viral titers of SARS-CoV-2 will be measured by quantitative PCR [50].

## Discussion

The rapid expansion of the COVID-19 pandemic has increased interest in finding safe and effective options for protecting healthcare workers and those at greatest risk of exposure.

PVP-I nasal spray and mouthwash could be considered a safe and potentially effective option against SARS-CoV-2 based on preliminary *in vitro* and *in vivo* studies cited in this review, although rigorous clinical trial data on SARS-CoV-2 are awaited to better understand the magnitude of effect and efficacy [16,68,69].

Tolerable commercial products containing saline that are used in nasal irrigation and as nasal sprays typically are at NaCl concentrations considered to be isotonic at 0.9% and hypertonic (up to 3.0%) [70]. The concentrations of NaCl mentioned in this review that have been investigated *in vitro* against SARS-CoV-2 and HCoV-229E range from 30 mM (0.17% NaCl) to 260 mM (1.5% NaCl). After irrigation with saline, most of the liquid will run out of the nose or mouth, removing excess mucous and leaving a thin film of salty water. The cells will then absorb chloride ion, so the concentration will change over time, and once the HOCl is used up, the virus will start to replicate again. Any NaCl concentration greater than 130 mM would result in a net increase in chloride ions adjacent to the nasal epithelium. A concentration of 260 mM NaCl (1.5%) shows the greatest inhibition of viral replication. Concentrations above 260 mM NaCl would be expected to suppress viral replication for a longer period of time. The increased chloride ion concentration in these cavities would cause the epithelial cells (where the virus replicates) to take up chloride, increasing the hypochlorous acid concentration through the activity of a peroxidase. This innate immune mechanism in epithelial cells may halt viral replication [32]. Additionally, increasing the NaCl concentration outside of the cells may result in the movement of water by osmosis out of the cells into the oral and nasal cavities which would have an added benefit of loosening mucous secretions and potentially easing some of the symptoms caused by SARS-CoV-2.

Listerine is a combination of up to 25% ethanol (at varying concentrations) plus essential oils that are both soothing and antimicrobial including eucalyptol, menthol, thymol and methyl salicylate [24]. The original Cool Mint Listerine has been reported to be similarly effective against SARS-CoV-2 *in vitro* as PVP-I [18]. In one preprint study, ethanol alone without essential oils at the concentrations found in Listerine at 21 and 23% *in vitro*, showed no virucidal activity indicating that the essential oils included are required for the virucidal activity observed [71]. This is consistent with data from one study that showed 20% ethanol decreased viral titers *in vitro* by 1.1log_10_, but increasing the ethanol concentration to ≥30% coincided with a decrease in viral titers by ≥5.9 log_10_ [17]. Another alcohol-based antiseptic, Nozin, has primarily been studied for decolonization of the nose to prevent *Staphylococcus* infections (MRSA, methicillin-susceptible *Staphylococcus aureus*), and while 60–70% is within the CDC-recommended alcohol content to kill most pathogens including viruses, no studies have investigated the efficacy of Nozin against specific viral infections [38]. It should also be noted that as an alcohol-based sanitizer, the duration of activity of Nozin is relatively short, and thus may need to be administered frequently for maximum effectiveness. Further study is warranted for alcohol and essential oil-based antiseptics against SARS-CoV-2.

Chlorhexidine is being evaluated in current clinical trials given its use as an oral mouthwash, its history as an antiseptic in surgery, and data supporting its possible effectiveness against SARS-CoV-2 [44,45,56]. In the study conducted in South Korea, there were only two patients and the viral load increased following the 2 h period of improvement [44]. This study was not placebo controlled so it is not clear whether chlorhexidine has a direct antiviral effect *in vivo* or if the reduction is just due to washing away viral particles without halting viral replication. This provides some support that Chlorhexidine could be a useful oral antiseptic against SARS-CoV-2 in the short term, but there needs to be a larger randomized placebo-controlled trial to elucidate whether chlorhexidine is suitable for widespread use. There are also several safety concerns such as concentration-dependent skin irritation, potential conjunctivitis, potential corneal damage if exposed to the eye, teeth staining and potential ototoxicity [72,73]. Chlorhexidine has also been reported to have a detrimental effect on a representative nitrate-reducing bacteria *V. dispar* of the oral microbiome indicating consistent use at high doses could have a negative effect on the flora of the oral cavity over time [24]. Given these concerns, chlorhexidine may be best used at a low concentration of 0.12%, such as those being evaluated in ongoing clinical trials.

Hydrogen peroxide has been used as an oral rinse for patients on ventilators to prevent ventilator-related pneumonia [47]. However, the *in vitro* data available for hydrogen peroxide suggests, it is less effective than PVP-I and Listerine in reducing the viral load of SARS-CoV-2 in the oral cavity [18]. One small study of hydrogen peroxide in ten patients reported no significant reduction of intraoral viral load in SARS-CoV-2 positive patients [48]. Hydrogen peroxide has been considered safe to use as a tooth-whitening treatment, but may cause tooth sensitivity and gingival irritation which is concentration dependent [74]. More research on the potential safety concerns of hydrogen peroxide may be required before it should be used as a widespread oral antiseptic. There are currently several clinical trials investigating some commercial hydrogen peroxide products that may be able to provide more information on the efficacy and safety of hydrogen peroxide as a means of preventing SARS-CoV-2 transmission [63].

Iota-carrageenan is nontoxic and works by creating a physical barrier against viruses in a nonclinical study of the compound [75]. The compound has also been reported to be effective against the common cold compared with placebo in clinical trials, making it a good potential candidate for reducing transmission of SARS-CoV-2 [76]. In an analysis of two randomized controlled studies of iota-carrageenan against the common cold, iota-carrageenan was shown to be more effective than placebo at reducing the duration of symptoms in patients infected with human coronavirus by 3.9 days on average in one trial (p < 0.01) and 3.1 days in the other trial (p < 0.01) [77]. Iota-carrageenan has been reported to be effective against SARS-CoV-2 *in vitro* alone and in combination with xylitol [19]. There currently is no published *in vivo* data, but iota-carrageenan is being investigated in a clinical trial as a preventative measure against COVID-19 [52].

Hypertonic saline halts replication inside the cell by increasing hypochlorous acid, so direct comparison with antiseptics PVP-I, Listerine, iota-carrageenan and chlorhexidine that have a direct effect on free virus *in vitro*, can be misleading. To date, hypertonic saline and iota-carrageenan are the only interventions that have been shown to reduce the duration of symptoms of coronaviruses in randomized controlled trials investigating the common cold [30,78–81]. Details of these randomized controlled trials can be found in Table 3. There are different mechanisms employed by each of the interventions mentioned in this review. One potential benefit besides the antiviral activity of these interventions is the mechanical effect of washing viral particles away, and frequency of use may be an important factor for success. There may be an advantage to combining or alternating between antiseptic and saline nasal and oral rinses in order to enhance results.

**Table 3.** Past randomized controlled trial results for nasal sprays and gargles against respiratory infections.

Clinical trial #/intervention	Use	Conc.	Intervention (n)	Placebo (n)	TSS_(2–4)_	Reduction in duration of illness vs control	p-value	Ref.
NCT02438579/hypertonic saline nasal irrigation and gargling	Six times per day for first 2 days, and reducing frequency on day 3 as symptoms improve	1.5–3.0%	30	31	N/A	1.9 days (95% CI = 0.4–3.3)	0.01	[78]
Eudract number 2007-007577-23/carrageenan nasal spray	One spray to each nostril three times per day for 4 days	Coldamaris nasal spray (0.12% Carrageenan	14	18	Intervention: 4.62 ± 2.06 Placebo: 6.28 ± 2.29	N/A	<0.046	[27]
ISRCTN52519535/carrageenan nasal spray	Three times per day for 7 days	Coldamaris nasal spray (0.12% carrageenan)	77	77	N/A	1.8 days	<0.038	[77,80]
ISRCTN80148028/carrageenan nasal spray	Three times per day for 7 days	Coldamaris nasal spray (0.12% carrageenan)	51	52	N/A	2.1 days	0.037	[77,81]
NCT01944631/carrageenan nasal spray^†^	One puff into each nostril four times per day for 4–10 days	1.20 g/l iota-carrageenan	97	97	Intervention: 5.67 placebo: 6.39	N/A	0.0364	[76,79]

† Analysis occurred after one patient was removed due to no respiratory viruses detected in nasopharyngeal samples.

TSS_(2–4)_: Mean total symptom score of 8 symptoms for day 2 through 4.

Clinical trials of other antimicrobial compounds include neem extract, beta-cyclodextrins, nitric oxide-releasing solutions (NORS), baby shampoo and intranasal heparin. Neem extract is a solution created from the ethanol extraction of the *Azadirachta indica* plant that is composed of many organic compounds that are reported to have antimicrobial, antifungal and antiviral properties [82,83]. Beta-cyclodextrins, commonly used to improve delivery of hydrophobic compound, are reported to have some antiviral properties [84]. NORS treatment may be useful due to nitric oxide having immunoregulatory and antiviral properties [85]. Heparin has been proposed as a potential therapeutic nasal rinse against COVID-19 as a potential antiviral based on the strong affinity that the SARS-CoV-2 spike protein has for heparin [86]. There is some evidence that baby shampoo is effective against HCoV-229E and is currently being evaluated [87]. Baby shampoo is composed of surfactants and has been investigated as an adjunct therapy in chronic rhinosinusitis [88]. The amphiphilic property of surfactants suggests that baby shampoo may be useful against enveloped viruses such as coronaviruses. Randomized clinical trials of nitric oxide-releasing solution SaNOtize and intranasal heparin are currently underway.

Most of the compounds described in this review have already been in widespread use, so safety concerns are minimal. However, further evaluation of the safety of these compounds should be conducted, to rule out potential issues that might include alteration of oral flora in the mouth and nasal passages, tooth staining, irritation of the epithelia, as well as potential anosomia (loss of smell).

There is sufficient evidence to justify further evaluation of mouthwashes (chlorhexidine, Listerine, hypertonic saline, hydrogen peroxide, iota-carrageenan) and nasal treatments (Nozin, PVP-I, chlorhexidine, hypertonic saline and iota-carrageenan) for reducing the rate of transmission; mitigation of disease progression by reduction of viral load very early after infection; and prophylaxis after exposure to SARS-CoV-2.

## Conclusion

While nasal sprays and gargles cannot replace the use of traditional personal protective equipment (i.e., gowns, masks, protective eyewear and gloves), the use of nasal and oral antisepsis has the potential to be useful for combating SARS-CoV-2. Clinical trials currently underway will be the best mechanism to determine the true benefit of these compounds and their role in mitigating disease progress and transmission of SARS-CoV-2 infection.

## Future perspective

Several of the compounds in this review may be commonly used against not only SARS-CoV-2 but also many other viruses given their broad-spectrum antimicrobial effects. The results of ongoing and future clinical trials will determine the role of these agents for SARS-CoV-2 mitigation. Given that these agents are broad-spectrum antiseptics rather than antibiotics, the risk of resistance over time may be low but should also be studied.

**Executive summary**

**Methods**

- A review of literature was conducted regarding the use of commercially available antiseptics and SARS-CoV-2.

**Results**

- Povidone-iodine (betadine), ethanol and essential oils (Listerine) and a combination of xylitol and iota-carrageenan (purified from red marine algae) were shown to reduce viral load of SARS-CoV-2 *in vitro* by 3–4 log_10_ in 30 s.
- Chlorhexidine, a widely used oral rinse, does not act as quickly in reducing viral load in 30 s as povidone-iodineI, but binds to cell proteins, extending protection.
- Hydrogen peroxide is not as effective as other oral rinses *in vitro* and cell toxicity is a concern.
- Hypertonic saline is not directly virucidal, but halts replication by increasing hypochlorous acid inside the cell.

**Conclusion**

- Several commonly used nasal antiseptics and gargles have shown efficacy against SARS-CoV-*2 in vitro* and clinical trials are currently underway to study their impact on disease course and transmission.

**Future perspective**

- These commercially available products should be further evaluated due to their potential ability to reduce the transmission of SARS-CoV-2 and other viruses that are yet to emerge.
